# Local Ablative Therapy in Breast Cancer Liver Metastases: Survival Outcomes and Prognostic Factors—A Single-Center Retrospective Study

**DOI:** 10.3390/jcm15114045

**Published:** 2026-05-23

**Authors:** Elif Sertesen Çamöz, Ahmet Bayrak, Cengiz Karaçin, Şahap Törenek, İlknur Deliktaş Onur, Tuğba Önder, Öztürk Ateş

**Affiliations:** 1 Department of Medical Oncology, University of Health Sciences, Dr. Abdurrahman Yurtaslan Ankara Oncology Training and Research Hospital, 06200 Ankara, Türkiye; cengizkaracin@yahoo.com (C.K.); ilknurdeliktas382@gmail.com (İ.D.O.); dr.ozturates@gmail.com (Ö.A.); 2 Department of Radiology, University of Health Sciences, Dr. Abdurrahman Yurtaslan Ankara Oncology Training and Research Hospital, 06200 Ankara, Türkiye; kaysbayrak@yahoo.com (A.B.); sahap.torenek@hotmail.com (Ş.T.)

**Keywords:** breast cancer, liver metastasis, microwave ablation, transarterial chemoembolization, local ablative therapy, survival, prognostic factors

## Abstract

**Background**: Liver metastases from breast cancer (BCLM) are associated with poor prognosis and represent a significant clinical challenge in the era of modern systemic therapies. Local ablative therapies (LATs), including microwave ablation (MWA) and transarterial chemoembolization (TACE), have emerged as potentially beneficial locoregional approaches in selected patients. However, data on survival outcomes and prognostic determinants of LAT in BCLM remain limited. This study aimed to evaluate the survival outcomes and prognostic factors of LAT in patients with breast cancer liver metastases at a tertiary care center. **Methods**: Patients with de novo or metachronous breast cancer liver metastases who underwent LAT (MWA and/or TACE) between 2013 and October 2023 at a single tertiary center were retrospectively analyzed. Primary endpoints were overall survival (OS), defined as the time from LAT initiation to death from any cause, and progression-free survival (PFS), defined as the time from LAT initiation to the first radiographically confirmed progression. Treatment response was assessed per RECIST 1.1 criteria. **Results**: A total of 20 female patients were included. Median age at diagnosis was 42 years (IQR: 37–53). The majority had invasive ductal carcinoma (90%) and grade 3 disease (60%). Hormone receptor-positive, HER2-positive, and triple-negative subtypes comprised 45%, 25%, and 30% of the cohort, respectively. MWA was performed in 16 patients (80%), TACE was performed in 2 patients (10%), and both modalities were performed in 2 patients (10%). Complete response per RECIST 1.1 was achieved in 40% of patients. No grade 3–4 adverse events were recorded. Median OS was 20 months (95% CI: 14.9–25.1), and median PFS was 6 months (95% CI: 0–17.5). In univariate analysis, factors associated with improved OS included LM size < 18 mm (23 vs. 11 months, *p* < 0.001), unilateral lobar involvement (23 vs. 5 months, *p* = 0.025), and LAT application during first-line therapy (48 vs. 19 months, *p* = 0.021). Factors associated with improved PFS included LM size < 18 mm (19 vs. 5 months, *p* < 0.001) and achievement of complete ablative response per RECIST 1.1 (18 vs. 5 months, *p* = 0.005). **Conclusions**: LAT is a safe and feasible treatment modality in selected BCLM patients. In univariate analysis, smaller lesion size, unilateral hepatic involvement, and early-line LAT applications are associated with improved OS, while complete ablative response is associated with improved PFS. These findings warrant validation in prospective studies with larger cohorts. Multidisciplinary patient selection is essential to optimize outcomes.

## 1. Introduction

Breast cancer is the leading cancer type in women worldwide and the second-leading cause of mortality [1]. Nearly 5–10% of breast cancer patients are de novo metastatic, and up to 30% of patients develop metastatic disease during their lifetime [2,3]. Despite advances in systemic therapies, particularly in the areas of targeted agents, immunotherapies, and antibody–drug conjugates (ADCs), metastatic breast cancer (MBC) remains a clinical entity that cannot be completely cured but is evolving into a chronic disease management process [2]. The liver is the third most common site for metastatic spread during the natural course of breast cancer, after the bone and lungs. The presence of liver involvement is an independent and strong negative prognostic factor that determines the course of the disease [4,5]. The highest incidence of liver metastasis is seen in HER2-positive and triple-negative disease; patients with triple-negative breast cancer develop liver metastasis within a median of 13–15 months of diagnosis [5]. Liver metastases (LMs) not only increase the tumor burden but also impair liver function, directly threatening the patient’s overall health and treatment tolerance [4,5].

The traditional oncological approach considered liver metastases as a harbinger of systemic disease and primarily limited treatment to systemic chemotherapy or endocrine therapies. However, the concept of “oligometastatic disease” [6] and the technological evolution of imaging-guided local ablative techniques (LATs) have led to a paradigm shift in the management of LM. The fundamental rationale for incorporating local approaches into systemic treatment in LM management is not only to reduce tumor burden but also to intervene in tumor biology. Achieving local control in liver-dominant progressive breast cancer offers advantages such as restoration of liver function, cessation of systemic treatment (chemo-holiday), combating chemoresistance, and immune modulation.

LAT modalities include surgical resection, thermal ablation methods such as radiofrequency ablation (RFA) and microwave ablation (MWA), stereotactic body radiotherapy (SBRT), transarterial chemoembolization (TACE), and combinations of these modalities [7]. Each method has different patient selection criteria and center experience, but generally, patients with a low metastatic burden (fewer than 3 tumors and smaller than 3 cm), hormone receptor-positive patients, patients with controlled extrahepatic disease, and patients with metachronous metastases benefit more from LAT [7]. In all cases, especially for patients diagnosed with liver metastatic breast carcinoma, treatment decisions should be evaluated in multidisciplinary councils at experienced centers [8].

In this study, we aimed to share our experiences with patients diagnosed with liver metastatic breast carcinoma who underwent LAT at a tertiary care center, and to identify prognostic factors influencing survival outcomes. Of note, while RFA-based data predominate in the BCLM ablation literature, MWA-specific series remain scarce; this study contributes real-world MWA-predominant data from a dedicated oncology center in the context of contemporary systemic therapy practice.

## 2. Materials and Methods

Patients with de novo or metachronous liver metastatic breast cancer, admitted to our center between 2013 and October 2023, who underwent microwave ablation (MWA) or transarterial chemoembolization (TACE) of liver metastases were retrospectively identified and included in the study. Inclusion criteria were: (1) histologically confirmed breast cancer with radiologically confirmed liver metastasis; (2) age ≥ 18 years; (3) Eastern Cooperative Oncology Group (ECOG) performance status 0–2 [9]; (4) at least one follow-up imaging examination after LAT; and (5) adequate hepatic function. Exclusion criteria were: (1) presence of uncontrolled extrahepatic disease at the time of LAT, defined as progressive disease at extrahepatic sites despite ongoing systemic therapy; (2) prior hepatic resection; and (3) incomplete clinical or follow-up data. No fixed institutional thresholds were applied for maximum lesion size or number; rather, eligibility for LAT was determined individually for each patient through multidisciplinary tumor board evaluation, taking into account lesion accessibility, expected technical success, hepatic functional reserve, overall disease burden, and the patient’s performance status. Hormone receptor status, HER2 status, and triple-negative status of breast cancer were recorded. Response to LAT was evaluated by the interventional radiologist who performed the procedure per Response Evaluation Criteria in Solid Tumors (RECIST) version 1.1 [10] on contrast-enhanced computed tomography (CT) or magnetic resonance imaging (MRI) performed at 4–8 weeks post-procedure and at subsequent 3-monthly intervals. Median follow-up was calculated from the date of LAT to the date of last follow-up or death. This study was reported in accordance with the Strengthening the Reporting of Observational Studies in Epidemiology (STROBE) guidelines [11]. This study was approved by the institutional ethics committee (Approval No: 2023-10/93) and conducted in accordance with the Declaration of Helsinki. Informed consent was obtained from all patients prior to the procedure.

The primary endpoints of the study were progression-free survival (PFS), defined as the time from LAT initiation to the first radiographically confirmed progression, and overall survival (OS), defined as the time from LAT initiation to death from any cause.

For the purpose of subgroup analysis, LAT timing was classified as “first-line” or “later-line” based on the line of metastatic systemic therapy at which LAT was administered. Patients who underwent LAT during their initial line of metastatic systemic therapy, including those with de novo metastatic disease who received LAT alongside their first systemic regimen, were categorized as “first-line LAT”. Patients who received LAT during their second or subsequent line of systemic therapy—that is, after progression on at least one prior metastatic regimen—were categorized as “later-line LAT”. The choice between first-line and later-line LAT was determined on a case-by-case basis through multidisciplinary tumor board discussion, considering the patient’s hepatic disease burden, performance status, prior treatment response, and overall disease trajectory.

The results were reported as mean ± SD, median, number (*n*), and percent (%). The Chi-square test compared categorical measurements. A *t*-test was used between independent groups. The Kaplan–Meier method was used to estimate the median PFS and OS. A log-rank test was used to compare the survival distributions between groups. A *p*-value < 0.05 was considered significant. The analysis was performed using SPSS v26.0.1.0 (IBM Corp., Armonk, NY, USA). Given the retrospective single-center design, no a priori sample size calculation was performed; instead, all consecutive patients meeting the inclusion criteria during the 2013–October 2023 study period were included. The resulting sample size (*n* = 20) reflects the real-world frequency of LAT utilization for BCLM at our institution. To contextualize the statistical power of the present analysis, a post hoc evaluation was conducted using the following assumptions: a two-sided alpha of 0.05, an observed event rate of 60% (12 deaths among 20 patients during follow-up), and approximately balanced subgroup distributions for the main stratification variables. With these parameters, the present sample provides adequate power for detecting large between-group differences in survival, as observed in the reported subgroup comparisons, but limited power to detect smaller effect sizes or to support multivariable modeling. The sample size, therefore, represents the principal constraint on statistical power for the analyses presented, and all observed associations are reported as hypothesis-generating, with effect-size interpretation taking precedence over formal hypothesis testing.

## 3. Results

A total of 20 female patients were included in the study. Baseline patient and treatment characteristics are summarized in Table 1. The median age at diagnosis was 42 years (IQR: 37–53). Eighteen patients (90.0%) had invasive ductal carcinoma, and 2 patients had invasive lobular carcinoma. Twelve patients (60.0%) had grade 3 differentiation. Nine patients (45.0%) had hormone receptor-positive disease, 5 (25.0%) had HER2-positive disease, and 6 (30.0%) had triple-negative disease. Three patients (15.0%) had de novo metastatic disease, and 17 (85.0%) had metachronous metastasis. The median time from diagnosis to liver metastasis was 36.5 months (IQR: 23.25–79.75). Four patients (20.0%) had a prior extrahepatic metastasis before liver involvement.

Microwave ablation (MWA) was performed in 16 patients (80.0%); 2 patients (10.0%) underwent TACE; and 2 patients (10.0%) underwent both modalities. A complete response per RECIST 1.1 was achieved in the liver lesion in 8 (40.0%) patients who underwent the procedure. The procedure was well tolerated overall. No grade 3 or 4 adverse events were recorded. Procedure-related complications occurred in 3 patients (15.0%) and were all classified as grade 1–2 per the Common Terminology Criteria for Adverse Events (CTCAEs): one case of post-procedural cholecystitis managed conservatively with intravenous antibiotics, intravenous fluid support, and bowel rest; one hepatic abscess that resolved with oral antibiotic therapy; and one episode of post-ablation fever that resolved spontaneously. No patient required additional procedural intervention, prolonged hospitalization beyond the planned admission, or discontinuation of subsequent systemic therapy due to LAT-related toxicity. The remaining 17 patients (85.0%) experienced no documented complications. The median LM size was 18 mm (95% CI: 16.5–30.9). The procedure was performed on 2 lesions in 5 patients (25.0%); the remaining patients had only 1 lesion. Bilateral lobar involvement was recorded in 3 patients (15.0%). Eight (40.0%) patients had the procedure during their second or later line of therapy. Five (25.0%) patients were identified as having local progression in the liver, while 15 (75.0%) patients first experienced systemic progression.

**Figure 1:**
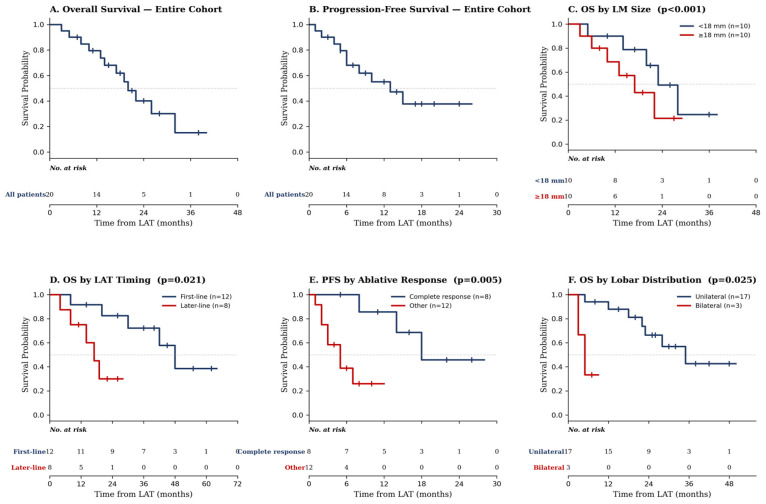
Kaplan–Meier curves for (**A**) overall survival (OS) and (**B**) progression-free survival (PFS) of the entire cohort, and OS/PFS stratified by (**C**) LM size, (**D**) LAT timing, (**E**) ablative response per RECIST 1.1, and (**F**) lobar distribution. Tick marks indicate censored observations. Numbers at risk are shown below each curve. LAT: local ablative therapy; LM: liver metastasis; CR: complete response.

At a median follow-up of 30 months (range: 4–86), the median OS was 20 months (95% CI: 14.9–25.1), with 1-year and 3-year OS rates of 73% and 36%, respectively. The median PFS was 6 months (95% CI: 0–17.5). In univariate analysis, factors associated with improved OS were LM size less than 18 mm (23 vs. 11 months, *p* < 0.001), unilateral lobar involvement (23 vs. 5 months, *p* = 0.025), and the use of LAT in first-line therapy (48 vs. 19 months, *p* = 0.021). Factors associated with improved PFS were LM size less than 18 mm (19 vs. 5 months, *p* < 0.001) and achievement of complete ablative response per RECIST 1.1 (18 vs. 5 months, *p* = 0.005). The limited sample size precluded multivariate Cox regression analysis. Detailed survival analyses are presented in Table 2, and Kaplan–Meier curves are shown in Figure 1.

## 4. Discussion

This single-center retrospective study evaluates the survival outcomes and prognostic factors of LAT in patients with breast cancer liver metastases. Our findings demonstrate that LAT is a safe and feasible approach in selected patients, with a median OS of 20 months and a median PFS of 6 months from the time of LAT initiation. In univariate analysis, smaller lesion size, unilateral hepatic involvement, and early-line LAT application were significantly associated with improved OS, while achievement of complete ablative response per RECIST 1.1 was associated with improved PFS.

Locoregional ablative therapies have emerged as an integral component of the multidisciplinary management of breast cancer liver metastases, offering a less invasive alternative to surgical resection in patients with limited hepatic disease who are not surgical candidates. Beyond local tumor control, these approaches may delay disease progression, preserve hepatic functional reserve, and allow continued systemic therapy in selected patients. Beyond breast cancer specifically, locoregional ablative therapies have demonstrated efficacy across various primary malignancies metastatic to the liver. In a series of 127 patients with unresectable colorectal liver metastases treated with percutaneous radiofrequency ablation, Facciorusso et al. reported favorable survival outcomes and identified the pre-treatment lymphocyte-to-monocyte ratio as an independent prognostic biomarker, illustrating the broader applicability of percutaneous ablation in metastatic liver disease and supporting the ongoing development of biomarker-driven patient selection [12].

The survival outcomes observed in our cohort are consistent with previously published data on locoregional therapies for BCLM. In a landmark series by Meloni et al., percutaneous radiofrequency ablation (RFA) achieved complete tumor necrosis in 97% of treated lesions, with a median OS of 29.9 months from the time of ablation [13]—a figure numerically higher than our cohort, potentially reflecting differences in patient selection, lesion size thresholds, and era-specific systemic therapy regimens. Bai et al. similarly reported a median OS of 26 months following percutaneous RFA and identified tumor size, ER positivity, and extrahepatic metastatic disease as independent prognostic factors for OS [14], findings largely concordant with our results. More recently, a 2025 single-center retrospective study evaluating percutaneous MWA and irreversible electroporation for BCLM reported favorable OS outcomes approaching those of surgical resection and SBRT in appropriately selected patients [15], further supporting the role of ablative approaches in this population.

The prognostic significance of LM size observed in our study aligns with the broader literature. Prior studies have demonstrated that metastatic lesions ≥ 2.5 cm carry a significantly worse prognosis following ablation, with hazard ratios exceeding 2.0 compared to smaller lesions [13,14]. Our analysis identified a median LM size of 18 mm as the critical threshold, below which both OS and PFS were substantially prolonged. This finding reinforces the importance of early identification and timely intervention before lesion enlargement compromises technical success and ablation margins.

A novel and clinically meaningful finding in our study is the significant association between LAT timing and OS. Patients who received LAT during first-line therapy achieved a markedly superior median OS of 48 months compared to 19 months in those treated in later lines (*p* = 0.021). To our knowledge, this is one of the few studies to directly compare survival outcomes by treatment line at the time of LAT in BCLM patients, and the magnitude of this difference—nearly 30 months—is clinically striking even in the context of a univariate analysis. This observation is biologically plausible: earlier LAT may interrupt the cycle of hepatic progression before the emergence of treatment-refractory clones, preserve liver functional reserve for continued systemic therapy exposure, and potentially engage immune-modulatory mechanisms at an earlier, more immunologically responsive disease stage. Supporting this, Glemarec et al. demonstrated in a series of oligometastatic breast cancer patients that LAT combined with systemic therapy yielded five-year OS rates of 63.2%, with LAT timing and hormone receptor positivity independently predicting both PFS and OS benefit [16]. Our results suggest that integrating LAT at earlier treatment lines—rather than reserving it as a salvage strategy for heavily pre-treated patients—may meaningfully improve outcomes and requires prospective evaluation in randomized or matched-cohort studies.

The clinical relevance of integrating LAT into systemic treatment regimens is increasingly supported by the comparative literature. In the series by Glemarec et al., oligometastatic breast cancer patients receiving combined LAT and systemic therapy achieved a five-year OS of 63.2%, which is substantially higher than historical controls treated with systemic therapy alone, with LAT timing emerging as an independent predictor of both PFS and OS [16]. Similarly, the MAMMA MIA registry demonstrated that the addition of locoregional ablative treatment to systemic therapy was associated with improved survival in carefully selected BCLM patients, with disease burden—rather than treatment modality alone—being the principal determinant of outcome [17]. Although our study lacks a systemic-therapy-only control arm and therefore cannot directly establish the additive benefit of LAT, the favorable median OS of 20 months observed in our cohort, particularly the 48-month median OS in first-line LAT recipients, is consistent with the survival advantage reported in these comparative series. Together, these data support the integration of LAT as a complementary, rather than alternative, therapeutic strategy alongside contemporary systemic regimens in selected BCLM patients.

Given the heterogeneity of metastatic distribution in our cohort, we performed a post hoc subgroup analysis to evaluate the influence of extrahepatic disease (EHD) on survival outcomes. Of the 17 patients with available data on metastatic distribution, 8 were diagnosed with disease confined to the liver and 9 had concurrent extrahepatic involvement (most commonly osseous, pulmonary, or both). Patients with liver-only disease demonstrated numerically superior survival outcomes compared to those with extrahepatic involvement, with a median OS of approximately 49 months versus 19 months and 3-year OS rates of 57% versus 25%, respectively. While this difference did not reach statistical significance (log-rank *p* = 0.346), likely due to the limited sample size in each subgroup, the magnitude of the observed difference is clinically meaningful and supports the hypothesis that LAT may confer particular benefit in patients with liver-confined oligometastatic disease—a population in whom achieving complete local control may translate into more durable disease control. This observation aligns with the broader oligometastatic paradigm [6] and warrants confirmation in larger prospective cohorts. Notably, this finding also suggests that the apparent OS benefit of first-line LAT observed in our cohort may be partially confounded by underlying differences in disease burden, as later-line patients more frequently presented with extrahepatic disease—an important consideration when interpreting subgroup associations in small retrospective series.

The safety profile of LAT in our cohort was favorable, with no grade 3 or 4 adverse events recorded. This is consistent with published complication rates for percutaneous ablative procedures, which are substantially lower than those associated with hepatic resection [7,18]. Patients with limited hepatic disease who are not surgical candidates are increasingly recognized as candidates for ablative therapies as part of a multimodal treatment approach [7], and our data contribute to the evidence base supporting this paradigm.

Notably, the type of progression following LAT—local versus systemic—did not significantly influence survival outcomes in our cohort (*p* = 0.469 for OS; *p* = 0.145 for PFS). The predominance of systemic progression (75%) over local recurrence (25%) suggests that LAT achieves adequate local control in most patients, and that overall disease trajectory is ultimately governed by systemic disease dynamics rather than the quality of locoregional treatment alone. This finding is consistent with the MAMMA MIA study, which similarly identified systemic disease burden rather than local control failure as the primary determinant of survival in locally ablated BCLM patients [17]. These data underscore the importance of concurrent effective systemic therapy alongside LAT.

This study has several important limitations that should be acknowledged upfront and considered when interpreting the findings. The two most significant limitations are the retrospective single-center design and the small sample size (n = 20), both of which constrain the generalizability and statistical robustness of our results. The retrospective design introduces inherent selection bias, as patients selected for LAT likely represented a more favorable performance status and disease burden than the broader BCLM population. The small sample size precluded multivariate Cox regression analysis; consequently, all survival comparisons are based on univariate log-rank tests and cannot establish independent prognostic associations after adjusting for confounders. The associations reported should therefore be interpreted as hypothesis-generating rather than confirmatory. Importantly, the heterogeneity of LAT modalities within the cohort represents a substantive methodological limitation. Although the cohort was predominantly treated with MWA (n = 16, 80%), the inclusion of patients who underwent TACE (n = 2) or combined MWA + TACE (n = 2) introduces inter-modality heterogeneity, as these techniques differ fundamentally in their mechanisms of action (thermal ablation vs. ischemic-cytotoxic effect), patient selection criteria, and technical success endpoints. Pooled analysis of these modalities may obscure modality-specific outcome patterns, and the small sample size precluded modality-stratified analyses. Future studies with larger, modality-homogeneous cohorts are needed to clarify the comparative effectiveness of each LAT approach in BCLM. Additional limitations include the absence of a control arm, which prevents causal inference regarding the survival benefit attributable to LAT; the lack of systematically captured data on concurrent systemic therapies and performance status at the time of LAT, which limited adjustment for these key confounders; and variability in systemic therapies across the study period (2013–2023)—spanning the introduction of CDK4/6 inhibitors, antibody–drug conjugates, and immune checkpoint inhibitors—which may have differentially influenced survival outcomes independent of LAT [2]. Despite these limitations, this study provides real-world data on MWA-predominant LAT in BCLM from a dedicated oncology center and generates clinically meaningful hypotheses regarding patient selection and LAT timing that warrant prospective validation.

## 5. Conclusions

In this single-center retrospective analysis of LAT in BCLM, smaller liver metastasis size, unilateral lobar involvement, and early-line LAT application were associated with improved OS in univariate analysis, while smaller lesion size and achievement of a complete ablative response per RECIST 1.1 were associated with improved PFS. The procedure was safe, with no grade 3–4 adverse events and no treatment-related discontinuations of subsequent systemic therapy. Although these findings are hypothesis-generating and require validation in larger prospective cohorts with multivariate adjustment, they collectively underscore that LAT candidacy in BCLM should not be determined by lesion characteristics alone, but rather through individualized assessment by a multidisciplinary tumor board that integrates lesion-related factors (size, distribution, and technical feasibility), patient-related factors (performance status, disease burden, and prior treatment exposure), and the broader treatment trajectory. Such a personalized, board-driven approach offers the best opportunity to identify patients most likely to derive durable benefit from LAT in the contemporary era of metastatic breast cancer management.

## Figures and Tables

**Table 1:** Baseline patient and treatment characteristics (n = 20).

Variable	Total (n = 20)
Patient characteristics	
Age at diagnosis, years—median (IQR)	42 (37–53)
Histological type—n (%)	
Invasive ductal carcinoma (IDC)	18 (90.0)
Invasive lobular carcinoma (ILC)	2 (10.0)
Tumor grade—n (%)	
Grade 1–2	8 (40.0)
Grade 3	12 (60.0)
Receptor subtype—n (%)	
Hormone receptor-positive (HR+/HER2−)	9 (45.0)
HER2-positive	5 (25.0)
Triple-negative (TNBC)	6 (30.0)
Metastasis pattern—n (%)	
De novo metastatic	3 (15.0)
Metachronous metastasis	17 (85.0)
Time from diagnosis to liver metastasis, months—median (IQR)	36.5 (23.25–79.75)
Prior extrahepatic metastasis before liver involvement—n (%)	4 (20.0)
Lat and lesion characteristics	
LAT modality—n (%)	
Microwave ablation (MWA) only	16 (80.0)
TACE only	2 (10.0)
MWA + TACE (combined)	2 (10.0)
LAT timing (treatment line)—n (%)	
First-line therapy	12 (60.0)
Second-line or later	8 (40.0)
Number of liver lesions treated—n (%)	
1 lesion	15 (75.0)
≥2 lesions	5 (25.0)
Median LM size at LAT, mm—median (95% CI)	18 (16.5–30.9)
Lobar distribution—n (%)	
Unilateral lobe involvement	17 (85.0)
Bilateral lobe involvement	3 (15.0)
Ablative response per RECIST 1.1—n (%)	
Complete response (CR)	8 (40.0)
Other (partial/stable/progressive)	12 (60.0)
Progression type after LAT—n (%)	
Local progression (hepatic)	5 (25.0)
Systemic progression	15 (75.0)

LAT: local ablative therapy; MWA: microwave ablation; TACE: transarterial chemoembolization; LM: liver metastasis; HR+: hormone receptor-positive; TNBC: triple-negative breast cancer; CR: complete response; IQR: interquartile range (Q1–Q3); CI: confidence interval. Prior extrahepatic metastasis was defined as any documented extrahepatic site of disease preceding the diagnosis of liver metastasis.

**Table 2:** Prognostic factors affecting median OS and median PFS following LAT.

Variable	Category	mOS (95% CI)	*p*-Value (OS)	mPFS (95% CI)	*p*-Value (PFS)
Number of LM			0.861		0.819
	1	20 (14.8–25.2)		6 (3.6–8.4)	
	2	17 (7.4–26.6)		12 (4.5–19.5)	
LM size			<0.001		<0.001
	<18 mm	23 (13.1–32.8)		19 (4.7–33.3)	
	≥18 mm	17 (9.9–24.0)		5 (2.9–7.0)	
LM distribution			0.025		0.356
	Unilateral lobe	23 (0–47.8)		12 (0.6–23.4)	
	Bilateral lobe	5 (NA)		5 (NA)	
LAT Timing			0.021		0.321
	First-line	48 (21.8–74.2)		15 (4.5–25.5)	
	Later-line	19 (11.3–26.5)		6 (2.8–9.2)	
LAT Response			0.156		0.005
	Complete response	48 (5.2–90.8)		18 (10.3–25.7)	
	Others	17 (0–35.7)		3 (0.9–5.1)	
Progression Type After LAT			0.469		0.145
	Local progression	23 (NA)		3 (NA)	
	Systemic progression	20 (13.0–26.9)		12 (2.7–21.3)	

mOS: median overall survival; mPFS: median progression-free survival; LM: liver metastasis; LAT: local ablative therapy; NA: not applicable; CI: confidence interval. All survival values are reported in months.

## Data Availability

The data that support the findings of this study are not publicly available due to patient privacy and confidentiality restrictions in accordance with institutional ethics committee requirements (protocol code 2023-10/93). The data may be made available from the corresponding author upon reasonable request, subject to appropriate data sharing agreements and institutional approval.
